# Robustness to Faults Promotes Evolvability: Insights from Evolving Digital Circuits

**DOI:** 10.1371/journal.pone.0158627

**Published:** 2016-07-13

**Authors:** Nicola Milano, Stefano Nolfi

**Affiliations:** 1 Institute of Cognitive Sciences and Technologies, National Research Council (CNR), Roma, Italia; 2 Center for Robotics and Neural Systems, Plymouth University, Plymouth, United Kingdom; Fred Hutchinson Cancer Research Center, UNITED STATES

## Abstract

We demonstrate how the need to cope with operational faults enables evolving circuits to find more fit solutions. The analysis of the results obtained in different experimental conditions indicates that, in absence of faults, evolution tends to select circuits that are small and have low phenotypic variability and evolvability. The need to face operation faults, instead, drives evolution toward the selection of larger circuits that are truly robust with respect to genetic variations and that have a greater level of phenotypic variability and evolvability. Overall our results indicate that the need to cope with operation faults leads to the selection of circuits that have a greater probability to generate better circuits as a result of genetic variation with respect to a control condition in which circuits are not subjected to faults.

## Introduction

Explaining how random genetic changes and natural selection can lead to complex useful innovation represents one of the hardest challenges for evolutionary theory. One crucial aspect behind innovation is constituted by the organism’s capacity to generate phenotypic variations in response to genetic and environmental variations. In that respect it is important to consider that phenotypic variation arises not only as a result of genetic variations but also as a result of environmental variations [1] and that the effect of genetic and environmental variations on the phenotype depends on the organization of the phenotype itself [1–3].

The objective of the work described in this paper is to verify experimentally whether the need to cope with environmental variations promotes evolvability, i.e. whether digital circuits evolved in variable environmental conditions discover more fit solutions than circuits evolved in stable environmental conditions. In particular, we investigate whether the need to cope with internal variation caused by component faults can promote evolvability.

We decided to use digital circuits since they have been widely used in artificial evolutionary studies [4–6] and since they share with natural systems (e.g. proteins, RNA, regulatory circuits and metabolic networks) the following properties [7–8]: (i) any phenotype (i.e. any circuit computing a given logic function) can originate from many different genotypes, (ii) these genotypes, giving rise to the same phenotype, can vary significantly among themselves, (iii) these genotypes span over vast genotype networks or neutral networks [9–10], i.e. genotypes giving rise to the same phenotype connected through single locus variation links, (iv) genotypes typically have many neighbors with the same phenotype and are thus robust to some extent to mutations, (v) the neighborhood of genotypes belonging to the same neutral network includes genotypes that give rise to rather different phenotypes.

For sake of clarity, we define phenotype as the logic function computed by a given circuit. We define *phenotypic variability* as the propensity of an individual or of a population to generate different unique phenotypes as a result of genetic variation. Variability should not be confused with variation, which refers to actual variations occurring between the individuals of a population (e.g. the heterozygosity or the degree of polymorphism of a population). Moreover we define the term *evolvability* as the propensity of genetic variations to sometime produce phenotypic adaptations. Phenotypic variability and evolvability indicate the potential or the propensity to vary and to improve, respectively, and “thus belong to the group of ‘dispositional’ concepts, such as solubility” [2]. Notice that although phenotypic variability likely correlates with evolvability, a high phenotypic variability does not necessarily imply a high evolvability. Multiple definitions of the term evolvability are in use [2–3, 10–11]. We adopted the definitions reported above since they enable us to distinguish between variations and adaptive variations and since they can be clearly operationalized in the case of our experiments. Indeed, they can be measured by generating from an evolving circuit a large number of genetically varied circuits and by counting the fraction of different unique phenotypes and the fraction of fitter phenotypes (for more details see below).

The relation between robustness to genetic variations, robustness to fault tolerance, and phenotypic variability in evolving digital circuits has already been investigated in several studies.

By exploring the space of 10^45^ logic circuits (genotypes) and of 10^19^ corresponding logic functions (phenotypes), Raman and Wagner [8] observed that: (i) the robustness of circuits with respect to mutation and to faults is high on the average, (ii) different circuits with the same phenotype have a broad distribution of robustness to genetic variations, with some circuits being much more robust than others, and (iii) larger circuits are more robust to mutations than smaller circuits. Larger circuits are more robust than smaller circuits also with respect to mutations that affect the components of the circuit that actively contribute to the output of the overall circuit. Moreover, the authors observed how neutral evolution tends to select circuits that have a high robustness with respect to mutation but a low phenotypic variability [8]. The neutral evolutionary process was realized by choosing a circuit computing a given function, generating an initial population composed of identical copies of the same circuit, generating varied copies of the circuits, and selecting the variations that preserve the function computed by the original chosen circuit.

Hu and all. [12–13], instead, analyzed in an exhaustive manner the space of 2^28^ logic circuits (genotypes) and of 16 corresponding logic functions (phenotypes). The circuits were constituted by 2 inputs, 4 gates and 1 output. Their analysis reveled that: (i) the genotype space is divided into only 16 fully connected neutral networks corresponding to 16 phenotype networks, (ii) the size of the networks varies significantly, and (iii) the genotypes located in the innermost core of the networks are characterized by a high level of robustness to genetic variation and by a low level of phenotypic variability. Moreover, by analyzing the course of random walk exploration processes carried out from a randomly selected genotype belonging to a given network toward the first encountered genotype belonging to a different network, the authors observed that: (i) different networks have rather different accessibility levels (i.e. probability to be reached through neutral and/or adaptive variations), (ii) the time necessary to reach a network through neutral and/or adaptive variations is correlated with the accessibility of the network (iii) the accessibility of a network is correlated with the robustness to genetic variations of the genotypes forming the network, and (iv) genotypes robust to genetic variations are more likely to be reached than less robust genotypes.

Finally, in a series of studies conducted by evolving digital circuits for the ability to perform a given target function, the authors demonstrated how forcing the circuits to operate in the presence of failures of circuit components lead to the evolution of circuits that are more robust against these faults [5, 14–15]. The robustness of evolving circuits is not achieved through the development of redundant solutions, i.e. solutions that include multiple copies of components and in which the failure of one component is compensated by the activity of another identical component. Rather robustness is achieved through degeneracy, i.e. through the ability of structurally different components to perform the same function [16–17].

In general terms, as discussed by Wagner (2008), the relationship between robustness to genetic variations and phenotypic variability is characterized by both antagonistic and synergetic factors. In fact, from the perspective of a specific genotype, the higher robustness to genetic variation of the genotype is, the lower phenotypic variability of the genotype is. On the other hand, from the perspective of a specific phenotype (i.e. from the perspective of the neutral network that includes all the genotypes connected through single genetic variations that give rise to the phenotype), the higher robustness of the genotypes forming the neutral network is, the higher the variety of the phenotypes that can be accessed in the neighborhood of the neutral network of the corresponding phenotype is [10]. Apparently, in some cases, the interplay of these factors can have an overall negative effect on phenotypic variability while in other cases it can have a positive effect. Indeed, by studying simulated RNA molecules that evolve toward a predefined target shape in a constant environment, Ancel and Fontana [18] observed a dramatic loss of variability throughout generations that ultimately traps populations in regions where most genetic variation is phenotypically neutral (a phenomena named “neutral confinement”). On the other hand, the systematic exploration of the genotype and phenotype space of simulated RNA molecules reported in Wagner [10] indicates that populations with robust phenotypes have a higher phenotypic variability than populations with less robust phenotypes.

In this paper we investigate the relation between environmental variations, phenotypic variability and evolvability. More specifically, we analyze whether the occurrence of faults drives the evolutionary process toward circuits with a high phenotypic variability and with a high evolvability. Moreover we analyze whether the occurrence of faults enables the evolutionary process to find more fit circuits with respect to a control condition in which circuits are not subjected to faults.

The results demonstrate that the need to cope with faults promotes the selection of phenotypically variable and evolvable circuits, and this, in turn, speeds up the evolution of effective circuits.

## Method

**Fig 1:**
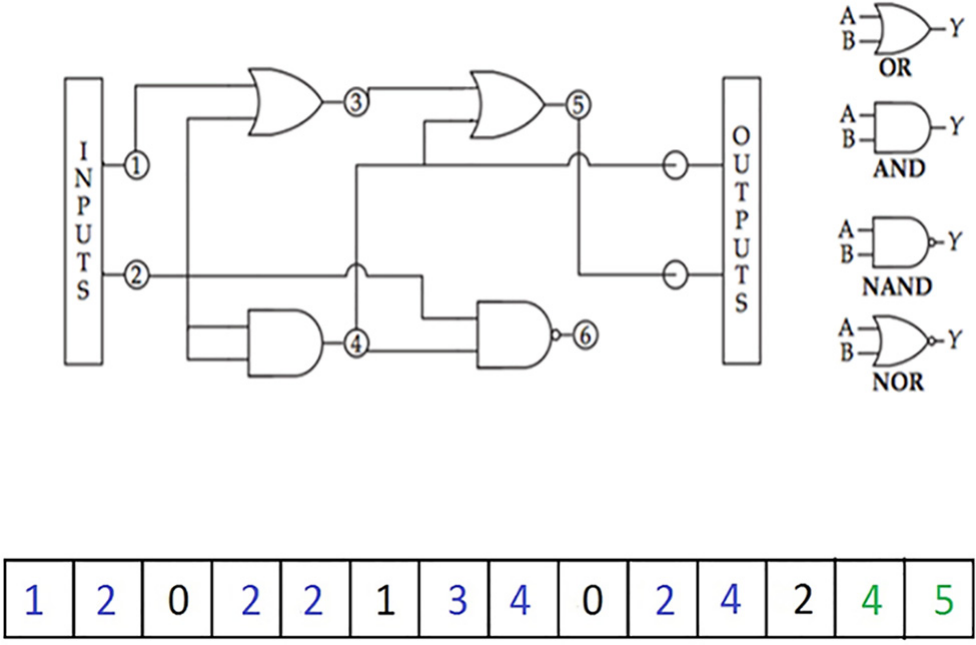
**Top**. A digital circuit with two inputs, two outputs, and four gates. The right side of the panel shows the four symbols that correspond to the four kinds of permissible logic gates. The numbers 1–2 indicate the binary states that are provided as inputs to the circuit (input pattern). The numbers 3–6 indicate the output computed by the four corresponding logic gates. The output of the circuit corresponds to the output of the two logic gates that are wired to the output pattern (4 and 5). The lines indicate the wiring of the circuit. **Bottom**. The genotype of the circuit shown on the top picture. The first 4 vectors of 3 numbers encode the characteristics of the four gates (from top to bottom and from left to right). The blue numbers encode the input states of each gate. The black numbers encode the function computed by each of the four gates (i.e. 0 = OR, 1 = AND, 2 = NAND, 3 = NOR). The last two numbers shown in green encode the output states of the circuit.

Digital circuits (Fig 1) are systems that compute logic functions, such as the multiplication of digital numbers, by receiving as input two or more binary (Boolean) values and by producing as output one or more binary values. They are composed of multiple logic gates that receive as input two binary values (from the input pattern and/or from the output of other logic gates) and produce as output one binary value by computing an elementary logic function (OR, AND, NAND ext.) of the input. The logic function computed by a circuit depends on the functions computed by its constituent logic gates and by the way in which they are wired.

Digital circuits can be realized in hardware or simulated in a computer. In standard electronic digital circuits the number and type of gates and the way in which they are wired is hardwired and hand-designed. In reconfigurable electronic digital circuits (such as the FPGA, see [19]), instead, the logic function computed by each gates and the way in which gates are wired can be varied. In evolvable hardware applications and in simulated evolving circuits the logic functions computed by each gates and the way in which gates are wired are encoded in artificial genotypes and evolved [4–6, 20]. Evolving circuits are selected on the basis of their fitness which is usually computed by measuring how well the function computed by a circuit approximates a given target function.

In our experiments we evolved simulated digital circuits with four inputs, 256 logic gates divided into 16 layers of 16 gates, and one output for the ability to compute a 4-bit even parity function (i.e. to produce as output 1 when there is an even numbers of 1 in the input pattern and 0 otherwise). We choose this function since it constitutes a rather difficult problem for evolving circuits including OR, AND, NAND, and NOR logic gates [6].

The genotype of evolving circuit is described by a vector of integer numbers that encodes the type of function computed by each logic gates and the way in which gates are wired (see Fig 1, bottom). This approach has been named Cartesian Genetic Programming [21]. More specifically each genotype includes 256*3 genes that specify the type and the inputs of each logic gate and 1 additional gene that specifies the ID number of the gate that determines the output of the entire circuit. The genes that encode the characteristic of each logic gate include three integers that represent the type of the logic gate and the ID number of the inputs of the gate. The values of the genes are bounded in the range {1, 4} in the case of integers encoding the type of the logic gate (1 = OR, 2 = AND, 3 = NAND, 4 = NOR), in the range {1, 4+(L-1)*16} in the case of the integers that encode the ID of the inputs of logic gates (where L is the layer of the corresponding logic gate), and in the range {5, 261} in the case of the gene that encodes the ID of the gate that constitutes the output of the entire circuit.

As in several related works [5, 8, 22], we choose to provide digital circuits with a fixed number of gates since this enables us to use a simple encoding schema. For alternative approaches in which the number of gates is variable see [15, 23]. However, notice that the usage of a fixed number of logic gates only limits the maximum size of the circuits. Indeed, as we will see, evolving circuits typically use a much smaller number of gates than the maximum number, i.e. they include several non-functional gates that do not contribute to the function computed by the circuit itself (for an example, see the gate computing the state 6 in Fig 1). In other words, the size of the evolving circuits varies in any case within the limit imposed by the maximum number of gates [20].

To evolve the circuits we created an initial population of 20 randomly generated genotypes encoding 20 corresponding digital circuits. Each circuit was allowed to produce an offspring, i.e. a mutated copy of the parent genotype in which genes were replaced with a certain probability (*MutRate*) with a number randomly generated with an uniform distribution in the appropriate range. Each mutant’s fitness was then compared to the lowest fitness in the parent population. If the mutant was better or equally good, it replaced the parent. The selection and reproduction process was repeated for a certain number of generations.

Circuits are evaluated for the ability to map the 2^n^ possible input patterns into the corresponding desired outputs (i.e. 1 for input patterns with an even number of 1 and 0 otherwise). More specifically the fitness is calculated on the basis of the following equation:

$$F=1-\frac{1}{2^{n}}\overset{2^{n}}{\underset{j=1}{\sum }}\left|O_{j}-E_{j}\right|\pm \varepsilon$$ (1)

Where *n* is the number of inputs of the circuit, *j* is the number of the input pattern varying in the range {1, 2^n^}, *O*_*j*_ is the output of the circuit for pattern *j*, *E*_*j*_ is the desired output for pattern *j* and ℇ is a noise value randomly selected in the range {-*Stochasticity*, *Stochasticity*} with a uniform distribution.

The function of noise is that to make the selection process probabilistic [24]. We decided to use this technique, rather than probabilistic selection operators such as roulette wheels or tournament selection, since its impact can be tuned quantitatively by varying a single parameter and since it is qualitatively similar to the stochastic variation of fitness caused by uncontrolled variations occurring in non-deterministic settings. As examples of non-deterministic setting consider the case of circuits subjected to random operation faults in which the effect of the faults is stronger or weaker depending on the malfunctioning gates or the case of evolutionary robotics experiments in which the fitness scored by a robot depends also on aspects that are variable (e.g. the initial position/orientation of the robot in the environment and/or the positions of the obstacles in the environment, see [25]).

In the fault experimental condition each logic gate fails with a certain probability, i.e. responds to its input by producing the wrong output. To promote the evolution of circuits robust to faults affecting different gates and combinations of gates, we evaluated the circuit for one trial without faults and for 200 trials with faults. Each trial includes all possible 2^n^ input patterns. The fitness is computed by averaging the fitness scored in the no-fault trial (calculated on the basis of Eq 1) and the average fitness scored on the worst trials subjected to faults:

$$Fitness=\frac{F_{n}+F_{f}}{2}$$ (2)

$$F_{f}=\frac{1}{WTrials}\overset{Wtrials}{\underset{j=1}{\sum }}F_{j}\;,\;WTrials\in \left[0,200\right]$$ (3)

Where *WTrials* is the number of worst trials considered to calculate the fitness scored on fault trials, *Fn* is the fitness scored during the no-fault trials calculated in the basis of Eq 1, *Ff* is average fitness scored during the worst fault-trials, and *200* is the total number of trials carried out with faults.

Finally, to promote the evolution of progressively more robust solutions, the probability *fr* that a logic gate undergoes to a fault (that is initially set to 0.3) is increased or decreased at the end of each generation when the average fitness scored during the worst trials is larger or smaller than ½ of the fitness scored in the no-fault trial, respectively:

$$fr+=fr\;*\;\left(\frac{F_{f}}{F_{n}}-\frac{1}{2}\right)/vFaultRate\;,\;0.04\leq fr\leq 0.8$$ (4)

Where *fr*, that is constrained in the range {0.04, 0.8}, is the probability that gates undergo to faults and *vFaultRate* is a constant that determines the variation rate of fr. The rationale behind this variation of the fault probability is that it enables to maintain a more constant level of variation to select upon. Comparative experiments performed by using constant fault probabilities led to worse performance (results not shown). The reason why we decided to use only the worst fault trials for the calculation of the fitness instead than all trials is that this increases the selective pressure toward the selection of circuits that are robust with respect to operation faults.

## Results

In this and in the following section we report the results obtained in the no-fault experimental condition. The results obtained by subjecting evolving circuits to faults are reported in section 3.2.

Readers might replicate all the experiments described in this paper by downloading and installing FARSA from “https://sourceforge.net/projects/farsa/” and the experimental plugin and configurations files from “http://laral.istc.cnr.it/res/digcir/digital-circuits-faults.zip”. Additional data and results not reported in the paper for reason of space can be downloaded from “http://laral.istc.cnr.it/res/digcir/faults-supplementary-matherial.zip”

**Fig 2:**
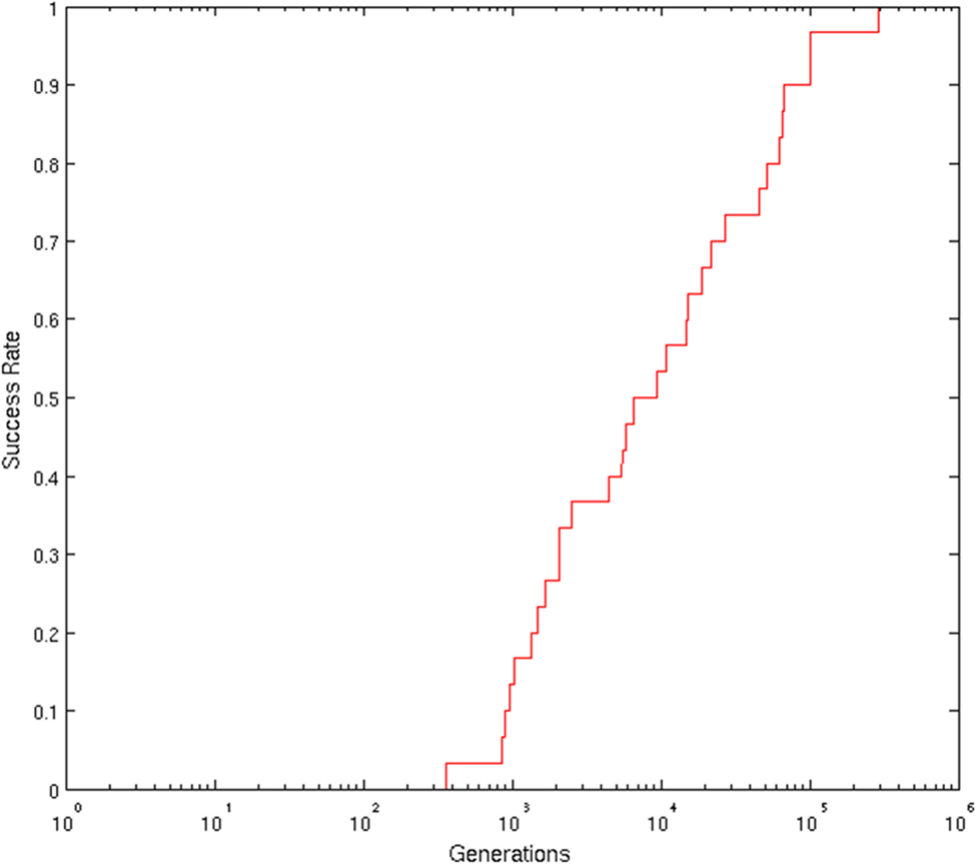
Percentage of evolutionary experiments that achieve maximum fitness through out generations in the no-fault condition. Data obtained by running 30 replications of the experiment for 300,000 generations with Stochasticity = 0.05 and MutRate = 0.02. Notice that the x-scale is semilogarithmic.

To verify the role of the mutation rate and of Stochasticity we measured the percentage of experiments that lead to optimal solutions within 6,000 generations for different values of the parameters (Table 1). As can be seen, in the case of the best combination of parameters (Stochasticity = 0.05 and MutRate = 0.02), evolving circuits find optimal solutions in 50% of the replications of the experiment. By continuing the evolutionary process for 300,000 generations (Fig 2) we can see how evolving circuits manage to achieve optimal performance sooner or later. However the discovery of optimal solutions might require a rather long evolutionary time, i.e. up to 300,000 generations.

**Table 1:** Percentage of evolutionary experiments that achieve maximum fitness in the no-fault condition in 20 experiments carried out with different mutation rate and stochasticity range. Each experiment has been replicated 30 times and continued for 6000 generations.

	MutRate 0.01	MutRate 0.02	MutRate 0.03	MutRate 0.04	MutRate 0.05
Stochasticity 0.0	26.66%	33.33%	33.33%	36.66%	36.66%
Stochasticity 0.05	33.33%	50%	46.67%	40%	36.66%
Stochasticity 0.08	26.66%	26.66%	33.33%	36.66%	30%
Stochasticity 0.1	20%	30%	30%	30%	26.66%

The data reported in Table 1 also show that the introduction of moderate level of noise in the selection process leads to better results (for similar results see [26,27,28]). This can be explained by considering that the addition of noise enables to select a limited number of less fit individuals, i.e. enable to reduce the selective pressure. This in turn increases the variation among the individuals of the population and reduces the risk of premature convergence.

### Evolution leads to the selection of small circuits with low evolvability

The analysis of evolved circuits in the no-fault condition indicates that, overall, they are very robust with respect to mutations. Indeed, by analyzing the effect of single point mutations we observed that the large majority of them are neutral (Table 2). This high robustness, however, is largely due to the fact that the size of the evolved functional circuit is small. Indeed by restricting the analysis to the mutations that affect the functional part of the circuits (i.e. to the mutations that alter the characteristics of the logic gates that actively contribute to the output of the overall circuit), the percentage of neutral mutations drops from 95% to 9%.

**Table 2:** Percentage of single point mutations that do not alter the fitness of the circuit. Data obtained by subjecting the best circuits of generation 6,000 to 10,000 single point mutations. The “functional” data refer to the mutations affecting the genes of functional gates (i.e. the gates that actively contribute to the output of the circuit). In this and in the following Tables/Figs of this section we refer to the 30 replications of the evolutionary experiment carried out in the no-fault condition with Stochasticity = 0.05 and MutRate = 0.02.

	% Neutral Mutations
Overall	95%
Functional	9%

The factor that drives evolution toward the selection of small circuits (i.e. circuit with a small functional part) is the fact that the smaller a circuit with respect to the other individuals of the population is, the larger the probability that its offspring will receive mutations that do not affect its functional gates, and consequently, the higher the probability that its offspring will have a relative higher fitness. This might present some similarities with the protection hypothesis that postulates that non-functional coding regions of the genotype might protect the evolving individuals from the deleterious effect of crossover [20].

**Fig 3:**
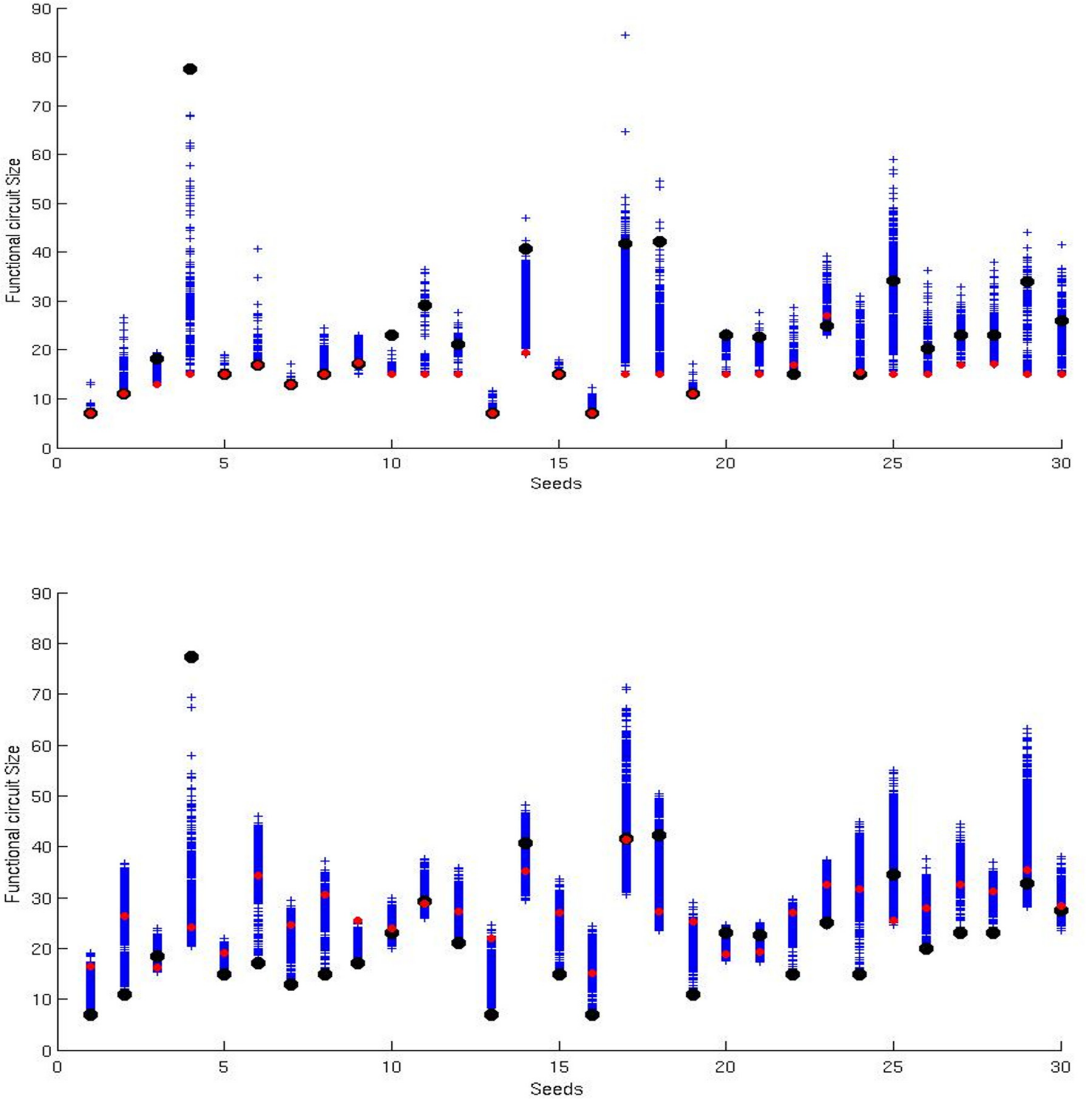
Variation of the functional size of evolved circuits while they are subjected to a neutral evolutionary phase for 100,000 generations. During the neutral phase, individuals are evaluated on the basis of a binary fitness function that assign a fitness of 1.0 to circuits that compute the same function of the initial circuits and a fitness of 0.0 otherwise. Circuits with fitness 1.0 and 0.0 are always selected or discarded, respectively. The black disks represent the average size of the evolved circuits before neutral evolution. The blue crosses indicate the average size every 100 generations during neutral evolution. Finally, the red disks represent the average size of circuits at the end of the neutral evolutionary phase. The top figure shows the results obtained in a normal condition in which selected offspring are used to replace one of the parents chosen randomly (all parents compute the same logic function and have the same fitness). The bottom figure shows the results obtained in a control condition in which offspring are used to replace their own parent. Data obtained by subjecting to neutral evolution 30 populations each composed of 20 circuits evolved for 6,000 generations.

This is demonstrated by the fact that the number of logic gates that actively contribute to the output of the circuit is only 30.4 and 18.3, on the average, in the case of circuits evolved for 6,000 generations displaying optimal or sub-optimal performance, respectively. Moreover, it is demonstrated by the fact that in most of the cases the size of the functional part of evolved circuits decreases through out generations, when they are subjected to a neutral evolutionary process in normal conditions (Fig 3, top, Wilcoxon Rank Sum Test, p < 0.001) while increases in a control condition in which selected offspring are used to replace only their own parent (Fig 3, bottom, Wilcoxon Rank Sum Test, p < 0.01). Notice that the latter condition corresponds to a situation in which the population is divided into 20 different single-individual sub-populations that evolve independently without competing with each other during selection. The tendency to select functionally small solutions, therefore, originates as a result of the selection process (only the best individuals reproduce) and as the result of the fact that smaller circuits have a greater probability to generate viable offspring than larger circuits.

**Fig 4:**
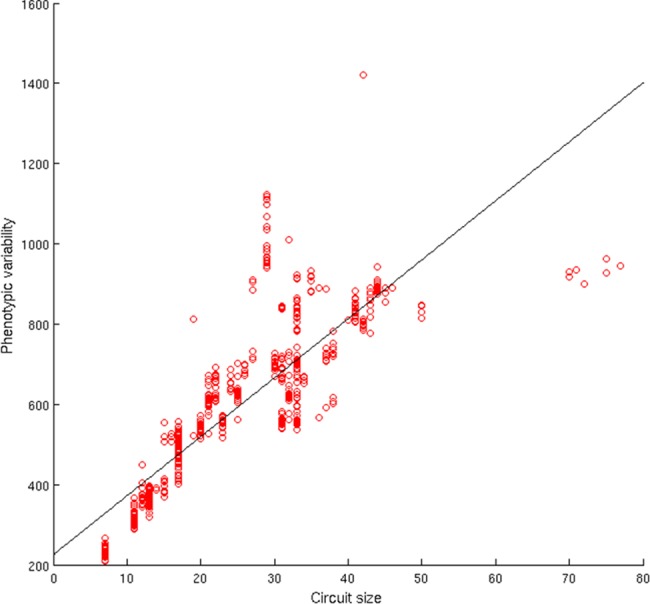
Distribution of the number of new unique functions found in the neighborhoods of evolved circuits and of the size of the circuits. Data obtained by analyzing the neighborhood of 600 evolved circuits obtained by running 30 evolutionary experiments for 6,000 generations. For each circuit, phenotypic variability has been calculated by counting the number of novel unique functions computed by sample circuits found in the neighborhood of the original circuit. Sample circuits have been selected by performing for 10 times a 1,000 steps function-preserving random walk from the original circuit. During each step we generated a varied circuit, through the application of a single point mutation, that was included in the sample set and used to generate additional varied circuits or discarded, depending on whether it computed the same function of the original circuit or not.

Unfortunately, however, the size of the evolving circuits strongly correlates with the phenotypic variability of the circuits, i.e. with the number of new unique phenotypes that can be found in the neighborhoods of the circuits (Fig 4, Spearman Test, rho 0.90229, phi 1.1153* 10^−220^ n = 600). This implies that evolution in normal conditions leads to the selection of functionally small circuits with low phenotypic variability and low evolvability (see also the results of the comparison with circuits evolved in the fault condition described below).

A correlation between circuit size and phenotypic variability has already been found by Raman and Wagner [8]. In their case the correlation was observed by comparing randomly generated circuits of different size that computed the same logic function. The correlation, therefore, seems to characterize all circuits, irrespectively from whether they were evolved or not and irrespectively from the function that they compute.

The fact that the growth of non-coding regions of the genotype can protect evolving individuals from deleterious genetic variations has already been pointed out in previous works [see 20]. Here we show that this protection can be achieved by shrinking the coding regions at the cost of a reduced phenotypic variability and evolvability.

**Fig 5:**
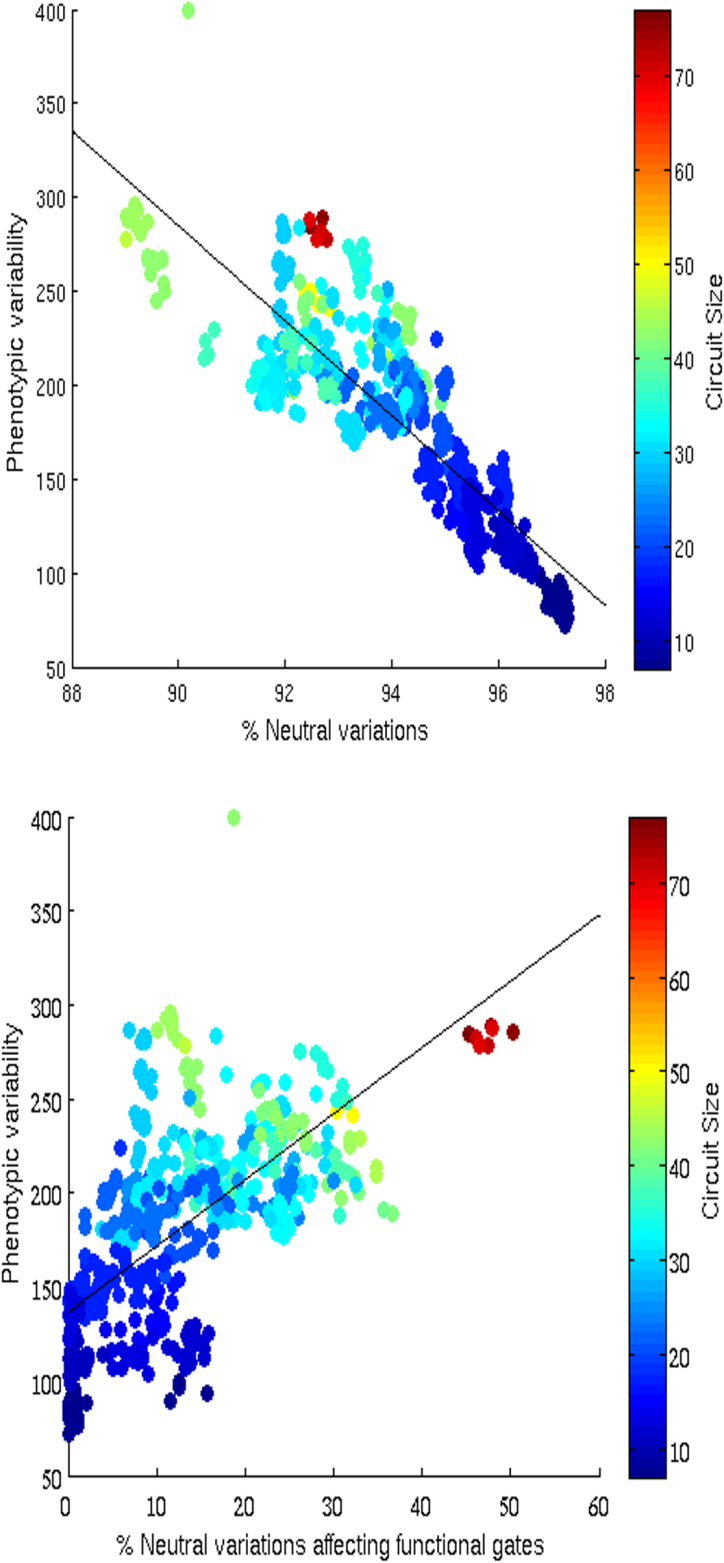
Distribution of phenotypic variability and robustness in evolving circuits. The top and the bottom Figures show the correlation between phenotypic variability and robustness to variations overall and to variations affecting the functional gates only, respectively. The color of each dot indicates the size of the corresponding circuit. Phenotype variability corresponds to the number of neighborhood circuits computing different unique functions and has been calculated by using the same procedure described in the caption of Fig 4.

The tendency of the population to move toward functionally small circuits during neutral evolutionary phases is a consequence of: (i) the fact that under neutral evolution the population tend to concentrate toward highly connected parts of the neutral network that correspond to individuals that are relatively robust against mutations [29–31], and (ii) the fact that individuals that are robust against mutations generally correspond to functionally small circuits (Fig 5, top).

The analysis of the relation between robustness to genetic variation and evolvability reveals a strong negative correlation with respect to overall variations (Fig 5, top, Spearman Test rho –0.87538, phi 6.4189* 10^−191^ n = 600) and a strong positive correlation with respect to variations affecting the functional components of the circuits only (Fig 5, bottom, Spearman Test rho 0.70301, phi 6.4189* 10^−191^ n = 600). This can be explained by considering that the circuits that are more robust to genetic variations overall are generally circuits with a small number of functional gates (see Fig 5). The robustness of these circuits with respect to genetic variations, therefore, is due primarily to their small size and not to a genuine ability to compensate the effects of variations. On the contrary, the circuits that are robust with respect to variations affecting their functional components are larger and are genuinely robust, i.e. are able to compensate to a greater extent the effects of genetic variations affecting functional gates (Fig 5, bottom). Larger circuits that are genuinely robust to genetic variations are also more evolvable with respect to smaller circuits that are robust only thanks to their small size.

### The need to face faults promote evolvability

In this section we report the results obtained by evolving circuits in the fault condition, i.e. in a condition in which each circuit gates is subjected to fault during operation with a certain probability.

To identify the optimal value of the two additional parameters that characterizes the evolutionary process in the fault condition we systematically varied the fraction of worst trials considered in the fitness function and the rate of variation of the fault frequency across generations. As shown in Table 3, the values of the parameters that maximize the percentage of replications leading to optimal performance in 6,000 generations are WTrials = 25% and vFaultRate = 5 (see Table 3). For the mutation rate we kept the same value that resulted optimal in the no-fault condition (MutRate = 0.02). The stochasticity range was set to 0.0 given that in the fault condition the fitness measure is already subjected to the stochastic effects caused by the randomly occurring faults, i.e. by the fact that the fitness loss caused by faults depends on the specific gates that are affected by faults. Indeed, in this case the usage of a stochasticity range greater than 0.0 is counter productive (see Table 4) and not beneficial as in the case of the no-fault condition (Table 1).

**Table 3:** Percentage of evolutionary experiments that achieved maximum fitness with different values of the vFaultRate and wTrial parameters. The MutRate and Stochasticity parameter are set to 0.02 and 0.0, respectively. Data obtained by running 30 replications, each lasting 6,000 generations, for each combination of parameters. The percentage of success refers to the performance achieved during the trial in which the circuits are not subjected to operational faults.

	vFaultRate = 1	vFaultRate = 3	vFaultRate = 5	vFaultRate = 7	vFaultRate = 9
WTrials = 35%	46.66	43.33	50	53.33	43.33
WTrials = 30%	50	50	**60**	53.33	40
WTrials = 25%	53.33	53.33	**60**	46.66	40
WTrials = 20%	50	46.66	53.33	40	36.66
WTrials = 15%	46.66	46.66	50	43.33	36.66
WTrials = 10%	33.33	30	33.33	30	26.66

**Table 4:** Percentage of evolutionary experiments that achieve maximum fitness in experiments carried out with different values of the Stochasticity parameter. Data obtained by running 30 replications lasting 6,000 generations for each value of the parameter. The Wtrial, vFaultRate, and MutRate parameters have been set 25%, 5, and 0.02, respectively. The percentage of success refers to the performance achieved during the trials in which the circuits are not subjected to operational faults.

Stochasticity = 0.0	Stochasticity = 0.01	Stochasticity = 0.02	Stochasticity = 0.03	Stochasticity = 0.04	Stochasticity = 0.05
60	36.66	30	33.33	23.33	20

The comparison of the results indicates that, as expected, the circuits evolved in the fault condition are more robust with respect to genetic variations affecting their functional components than the circuits evolved in the no-fault condition (Table 5). Indeed, the percentage of variations affecting functional gates that do not produce any loss in performance is 2.05% and 1.01% on the average, in the case of fault and no-fault circuits, respectively. The circuits evolved in the no-fault conditions are more robust with respect to overall variations than the circuits evolved in the fault condition. As discussed above, however, this does not reflect a genuine robustness but simply the fact that circuits evolved in the no-fault conditions are smaller than the circuits evolved in the fault condition.

**Table 5:** Characteristics of the neighborhoods of circuits evolved in the no-fault and fault conditions. Functional variations refer to variations affecting the gates that actively contribute to the output of the circuits. Data obtained by analyzing 600 evolved circuits obtained by running 30 evolutionary experiments for 6000 generations in each condition. Each circuit was subjected for 100 times to a 1,000 steps function-preserving random walk (see caption of Fig 4).

	No-fault	Fault	p-value (Wilcoxon Rank Sum test)
% neutral variations	94.2601	89.3427	<10^−132^
% maladaptive variations	5.7389	10.6559	< 10^−136^
% adaptive variations (evolvability)	0.0010	0.0014	< 10^−5^
% functional neutral variations	0.9986	2.0705	< 10^−53^
% functional maladaptive variations	4.0953	7.6177	<10^−142^
% functional adaptive variations	0.0008	0.0011	< 10^−4^
number of new unique phenotypes (phenotypic variability)	583.42	914.80	<10^−98^

**Fig 6:**
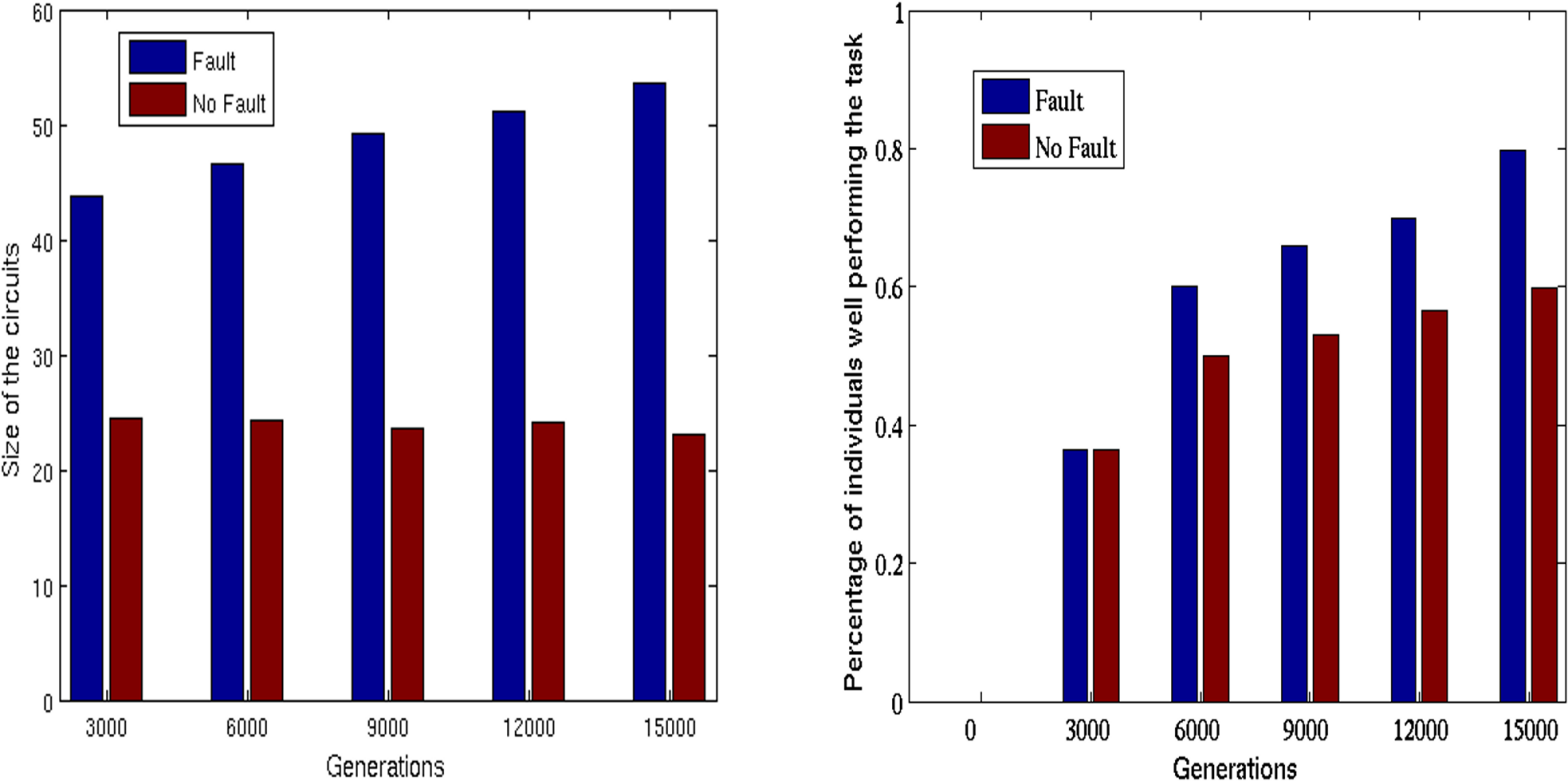
Left: Average size of functional circuits throughout generations in the no-fault and fault conditions. Right: Fraction of replications achieving optimal performance throughout generations in the no-fault and fault conditions. Data obtained by running 30 replications in each condition for 15,000 generations with the best parameters (i.e. Mutrate = 0.02 and Stochasticity = 0.05 in the no-fault condition; Mutrate = 0.02, stochasticity = 0.0, Wtrial = 25% and vFaultRate = 5, in the fault condition). The experiments conducted in the fault condition are considered successful when the evolving circuits display optimal performance during the trial in which the circuit component are not subjected to fault. In other words, the data displayed in the right Figure are not influenced by the level of robustness of the circuits with respect to operational faults. Wilcoxon rank sum tests p-value < 10^−63^.

The circuits evolved in the fault condition are larger than those evolved in the no-fault condition from generation 6,000 on (see Fig 6, left).

The circuits evolved in the fault condition have a greater phenotypic variability (Table 5).

Finally, the circuits evolved in the fault condition have a greater evolvability. This is demonstrated both by the fact that the percentage of genetic variations leading to improvements is higher in the case of the circuits evolved in the fault condition (Table 5) and by the fact that the circuits evolved in the fault condition achieve better performance from generation 6,000 on with respect to circuits evolved in the no-fault condition (Fig 6, right). As expected the probability that random genetic variations lead to improvement (evolvability) is rather low in both cases (Table 5). However it is higher for circuit evolved in the fault than in the no-fault condition. Notice that the evolvability is necessarily 0 for circuits displaying optimal performance. Since the number of optimal circuits evolved in the fault condition is greater than the number of optimal circuits evolved in the no-fault condition, the difference in evolvability between the two conditions is even greater than that reported in Table 5. This strengthens our conclusion that circuits evolved in the fault condition have a greater evolvability than circuits evolved in the no-fault condition.

**Fig 7:**
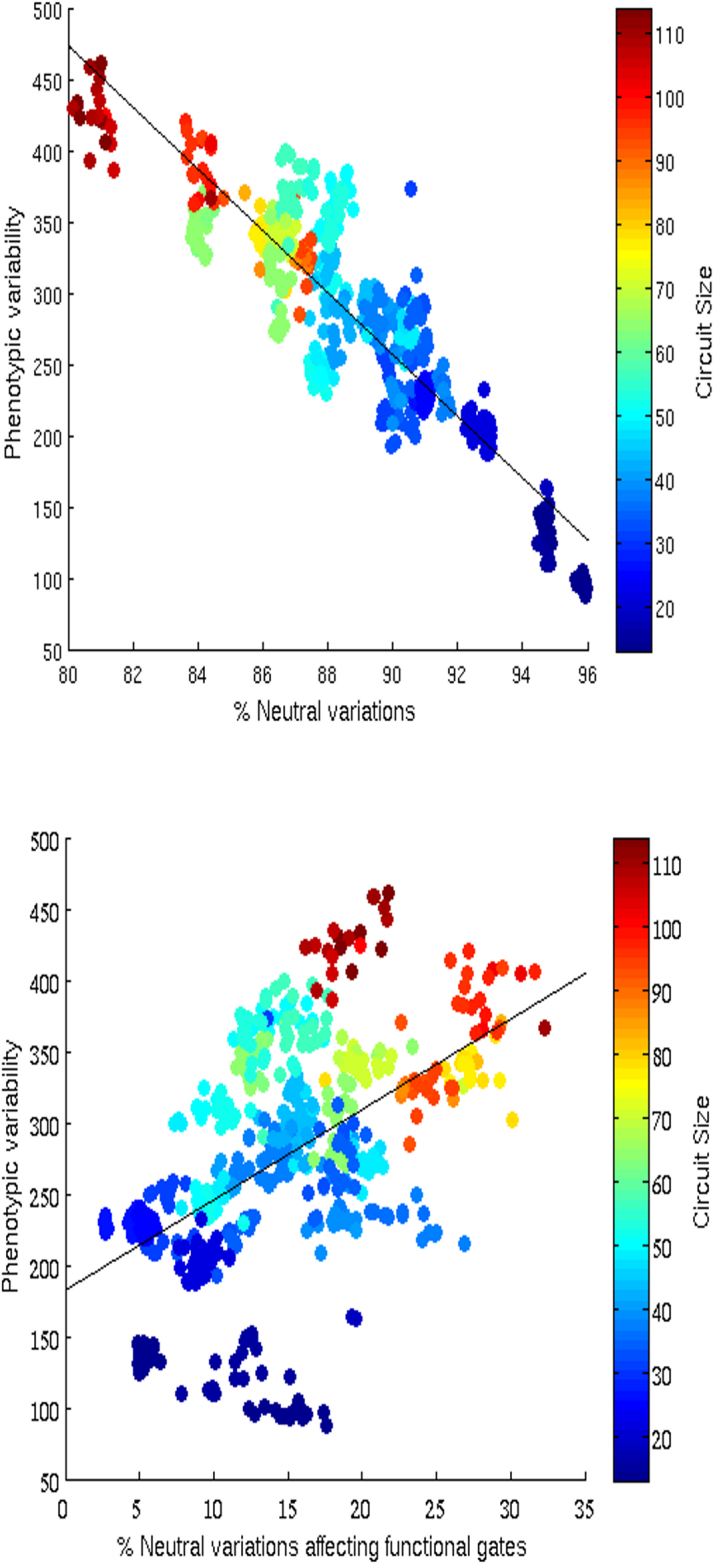
Distribution of phenotypic variability and robustness in evolving circuits. The top and the bottom Figures show the correlation between phenotypic variability and robustness to variations overall and to variations affecting the functional gates only, respectively. The color of each dot indicates the size of the corresponding circuit. Data obtained by analyzing the neighborhood of 600 evolved circuits obtained by running 30 evolutionary experiments for 6,000 generations. Phenotypic variability has been calculated by using the same procedure described in the caption of Fig 4.

The analysis of the relation between robustness to genetic variations and phenotypic variability reveals, also in this case, a strong negative correlation with respect to overall variations (Fig 7, top, Spearman Test rho –0.8679, phi 7.2153* 10^−184^ n = 600) and a positive correlation with respect to variations affecting the functional gates of the circuits only (Fig 7, bottom, Spearman Test rho 0.54292, phi 2.6872* 10^−47^ n = 600).

## Conclusions

In this paper we investigated whether the need to cope with component faults promotes evolvability in evolving digital circuits. To verify this hypothesis we compared the results obtained in two series of experiments in which the evolving circuits were subjected or not subjected to faults, respectively. The evolvability of the circuit was measured both directly, by calculating the fraction of variations that lead to improvements in evolving circuits, and indirectly, by verifying whether evolving circuits subjected to fault were able to achieve better performance in a given number of generations. The data collected demonstrate that indeed circuits subjected to fault are more evolvable than the circuits that are not subjected to faults, with respect to both direct and indirect measures of evolvability (see Fig 5 right and Table 5). As far as we know, this is the first time that this effect is demonstrated through artificial evolutionary experiments. Moreover, the analysis of our results indicates why the need to cope with faults promotes evolvability.

The analysis of the circuits evolved in the no-fault condition indicates that they tend to be very robust with respect to genetic variations. Indeed, the large majority (95%) of the genetic variations affecting evolved circuits are neutral, i.e. do not alter the fitness of the circuits. This is due to the fact that mutations enhancing robustness with respect to genetic variation tends to be retained in further generations even if they are adaptively neutral (i.e. even if they do not cause any fitness improvement). This in turn is due to the fact that the offspring of individuals that are more robust to genetic variations, with respect to the other individuals of the population, have a lower probability to generate offspring affected by maladaptive mutations.

The tendency to select individuals that are robust with respect to genetic variations have both negative and positive effects [32]. The negative effect arises as a consequence of the fact that the robustness with respect to genetic variations is negatively correlated with phenotypic variability, i.e. with the number of different unique phenotypes that can be generated by mutating an individual genotype. The positive effect arises as a consequence of the fact that the robustness with respect to genetic variations enables the evolving population to retain a higher number of (neutral) variations that in turn enable the population to explore a larger portion of the neutral networks on which the evolving individuals are currently located. As claimed by Wagner (2008), the net effect of these negative and positive effects can be positive. This can be explained by considering that the increase in phenotypic variability gained from the population thanks to its enhanced ability to retain genetic variations is greater than the loss of phenotypic variability occurring at the level of the single individuals. This in turn can be explained by considering that what matters from the point of view of innovating is the possibility to access to new phenotypes not accessed before and that the neighborhoods of individuals located in different areas of the same neural networks tend to include different unique phenotypes [8, 10].

Unfortunately, however, the selection of individuals robust with respect to genetic variation drives the evolutionary process toward the synthesis of minimal solutions, i.e. toward circuits that operate on the basis of a minimum number of functional logic gates (see Figs 3 and 6). In other words, it drives the evolutionary search toward a specific subarea of the neutral network. This might reduce and/or eliminate the advantage that can be gained by exploring different areas of the neutral networks, i.e. the advantage deriving from the fact that different areas of the neutral networks tend to include different unique phenotypes in their neighborhoods. Moreover, due to the negative correlation between robustness to genetic variation and phenotypic variability (Fig 5 top, [8]), the tendency to maximize robustness by selecting minimal solutions drives the evolving population toward a sub-area of the neutral network that is characterized by a low phenotypic variability. This implies that the selection of individuals that are robust with respect to mutations might drive the evolutionary process toward low evolvable solutions and eventually to evolutionary stagnation. Overall this implies that robustness to genetic variations achieved in this way, i.e. achieved by simply minimizing the number of genes encoding functional traits, does not promote but rather reduces evolvability. These minimal circuits are actually not truly robust with respect to genetic variations. They are not able to tolerate genetic variations through redundancy or degeneracy.

Instead, the need to cope with faults drives the evolutionary process toward the selection of circuits that are not only robust against operation faults (as shown in [5, 14–15]) but that are also genuinely robust against genetic variations (i.e. that are able to tolerate a greater number of variations affecting their functional components with respect to circuits evolved in the no-fault condition, see Table 5). The synthesis of circuits characterized by this form of robustness reduces the tendency to select minimal solutions (Fig 6) and promotes the selection of solutions characterized by higher phenotypic variability (Fig 7 and Table 5) and higher evolvability (Table 5). In other words it enhances the probability to generate new better phenotypes as a result of genetic variation both from the perspective of the single individuals and from the perspective of the population that is no more constrained toward the specific sub-area of the neutral network containing small circuits.

Robustness to operational faults constitutes probably the simplest form of environmental variation that one can study. In future research we plan to investigate the relation between evolvability and other form of environmental variations, e.g. systematic non-random variation of the external environment. Future work should also investigate the effect of operation faults in experiments in which variations are introduced also through crossing over, duplication, and deletion genetic operators.
